# Crimean–Congo Hemorrhagic Fever Outbreak in Podor, Northern Senegal in 2022: Two Independent Emergences and Unprecedented Mortality

**DOI:** 10.4269/ajtmh.24-0445

**Published:** 2025-07-08

**Authors:** Ousseynou Sene, Samba Niang Sagne, Déthié Ngom, Mamoudou Bocoum, Mamadou Korka Diallo, Aliou Khoulé, Elisabeth Thérèse Faye, Sokhna Mayemouna Diop, Idrissa Diallo, Cheikh Loucoubar, Mawlouth Diallo, Ndongo Dia, Yoro Sall, Boly Diop, Mamadou Ndiaye, Amadou Alpha Sall, Manfred Weidmann, Moussa Moise Diagne, Malick Fall, Ousmane Faye, Oumar Faye, Etienne Simon-Lorière, Diawo Diallo, Mamadou Aliou Barry, Gamou Fall

**Affiliations:** 1 Virology Department, Institut Pasteur de Dakar, Dakar, Senegal;; 2 Epidemiology, Clinical Research and Data Science Department, Institut Pasteur de Dakar, Dakar, Senegal;; 3 Zoology Department, Institut Pasteur de Dakar, Dakar, Senegal;; 4 Saint-Louis Regional Health Department, Podor Health District, Podor, Senegal;; 5 Quality Management Division, National Hygiene Service, Podor, Senegal;; 6 Prevention Department, Ministère de la Santé et de l’Action Sociale, Dakar, Senegal;; 7 Institute of Microbiology and Virology, Brandenburg Medical School, Brandenburg, Germany;; 8 Animal Biology Department, Faculty of Sciences and Techniques, Cheikh Anta Diop University, Dakar, Senegal;; 9 G5 Evolutionary Genomics of RNA Viruses, Institut Pasteur de Paris, Paris, France

## Abstract

Crimean–Congo hemorrhagic fever (CCHF) is a lethal zoonotic disease transmitted through tick bites and contact with infected animals or humans. As CCHF continues to expand worldwide, we report on the first severe outbreak in Senegal (Podor, Saint-Louis region) in 2022. We conducted a comprehensive outbreak investigation after a confirmed CCHF human case in Podor. This included sample collections from humans, animals, and ticks from the household and surrounding area. Human and animal samples were tested by ELISA for antibodies to CCHF virus and by reverse transcription polymerase chain reaction (RT-PCR) for CCHF virus RNA, whereas tick samples underwent CCHF RT-PCR only. Positive RT-PCR samples underwent viral genome sequencing for genetic characterization. We determined three CCHF human cases, with two deaths, as well as virus circulation in 11 ticks and in livestock, with an overall seroprevalence of 42.5%. CCHF IgG antibodies were detected in human contact cases, showing its prior prevalence in the area. Phylogenetic analyses revealed high genetic diversity within CCHF genotypes, with existence of reassortants and cocirculation of two different isolates from various origins in Mboyo and Nenette villages, where human cases were detected. The data showed a correlation between the strains identified in humans and ticks in each village, showing two independent emergences in Podor. The outbreak in Podor was probably because of the high abundance of animal hosts in this sylvopastoral area, the diversity of tick populations with the presence of the main CCHF vectors, and the increasing prevalence of CCHF virus. The surveillance in this area needs to be strengthened.

## INTRODUCTION

Crimean–Congo hemorrhagic fever (CCHF) is a severe and sometimes lethal infectious disease of humans described in more than 50 countries across Eurasia and Africa, where it is associated with sporadic cases and outbreaks of hemorrhagic fever or sporadic cases.^1^ The causative agent (Crimean–Congo hemorrhagic fever virus [CCHFV]) is an enveloped virus with three negative-sense genome segments: short (S), medium (M), and long (L).^2^ The virus has six distinct genetic lineages termed genotypes: I–III (in Africa), IV (in Asia), and V and VI (in Europe).^3^ CCHFV belongs to the genus Orthonairovirus of the *Nairoviridae* family in the Bunyavirales order.^4^ CCHFV is maintained in nature through both vertical and horizontal transmission in several genera of hard ticks (ixodid), which is conductive to the virus spreading to a variety of wild and domestic animals that develop transient viremia associated with an asymptomatic form of the infection.^5^ Humans are infected by bites from infected ticks, mainly of the *Hyalomma* genus, and by contact with the blood or tissues of viremic livestock or patient material during the acute phase of the infection.^6^ Humans are the only known hosts of CCHFV who develop symptomatic disease. CCHF patients usually experience a short incubation period (3–7 days) and then, develop clinical symptoms, including sudden onset of fever associated with fatigue, muscle pains, oliguria, and disturbance of consciousness.^7,8^ In severe cases, hemorrhagic manifestations can develop several days after the onset of the disease.^2^ Documented case fatality rates can range from 9% to 50% in hospitalized patients.^9^

The (re-)emergence of the virus continues to be a key topic for national and international health security, and CCHF is classified by the WHO as a priority disease for research and development because of its epidemic potential, high mortality rate, possibilities of nosocomial infection, and difficulties in treatment and prevention.^10–12^ Moreover, the isolation of the virus is very restrictive because it must be carried out in biosafety-level 4 laboratories (BSL4).^2^ Diagnostics, however, can be performed at BSL2.^13^

In Africa, a total of 494 CCHF human cases and 115 deaths were reported between January 1956 and July 2020.^14^ Since 2020, nine African countries (Kenya, Mali, Mozambique, Nigeria, Senegal, Sierra Leone, South Sudan, Sudan, and Tunisia) have documented CCHF cases.^14^ Mauritania, a country bordering Senegal, reported the first human case of CCHF in 1983.^15^ Since then, multiple severe CCHF outbreaks have been documented in Mauritania, whereas only a few sporadic cases and no mortalities have been reported from Senegal.^16–18^ Indeed, only one CCHF case was exported from Senegal to France, and the patient, who ultimately died, has been reported.^19^

Previous research activities indicated that the virus is highly prevalent in northern Senegal at the border with Mauritania, circulating in ruminants and ticks and determined by several virus isolations.^20,21^ Recent studies performed in northern Senegal have also reported a high seroprevalence in animals, a human case, and strains from ticks belonging to different genotypes.^17,22^ All of these data confirm that northern Senegal is at high risk of CCHF emergence. Considering this high risk of CCHF emergence in northern Senegal and the existence of multiple CCHF genotypes, reliable and up-to-date surveillance systems are needed to accurately assess the incidence and spread of CCHFV in this area as well as in other Senegalese regions. A severe CCHF outbreak occurred in the Podor region, northern Senegal (Mboyo and Nenette villages) in 2022, with two mortalities of three confirmed human cases. Outbreak investigations in humans, ticks, and livestock were performed. The first one was performed in Mboyo village after the occurrence of the index case, whereas a second one involving more field teams and more villages (Mboyo, Nenette, and other surrounding villages) was performed after the occurrence of the second CCHF case in Mboyo. The results of the second outbreak investigations were published by our team and described the prevalence of the virus in livestock and ticks in the area, the associated risk factors, and the epidemiological implications.^23^ Here, we describe the human CCHF cases, the global prevalence in ticks and animals, and the genetic characterization of the viral strains isolated in humans and ticks.

## MATERIALS AND METHODS

### Study area.

**Figure 1.**
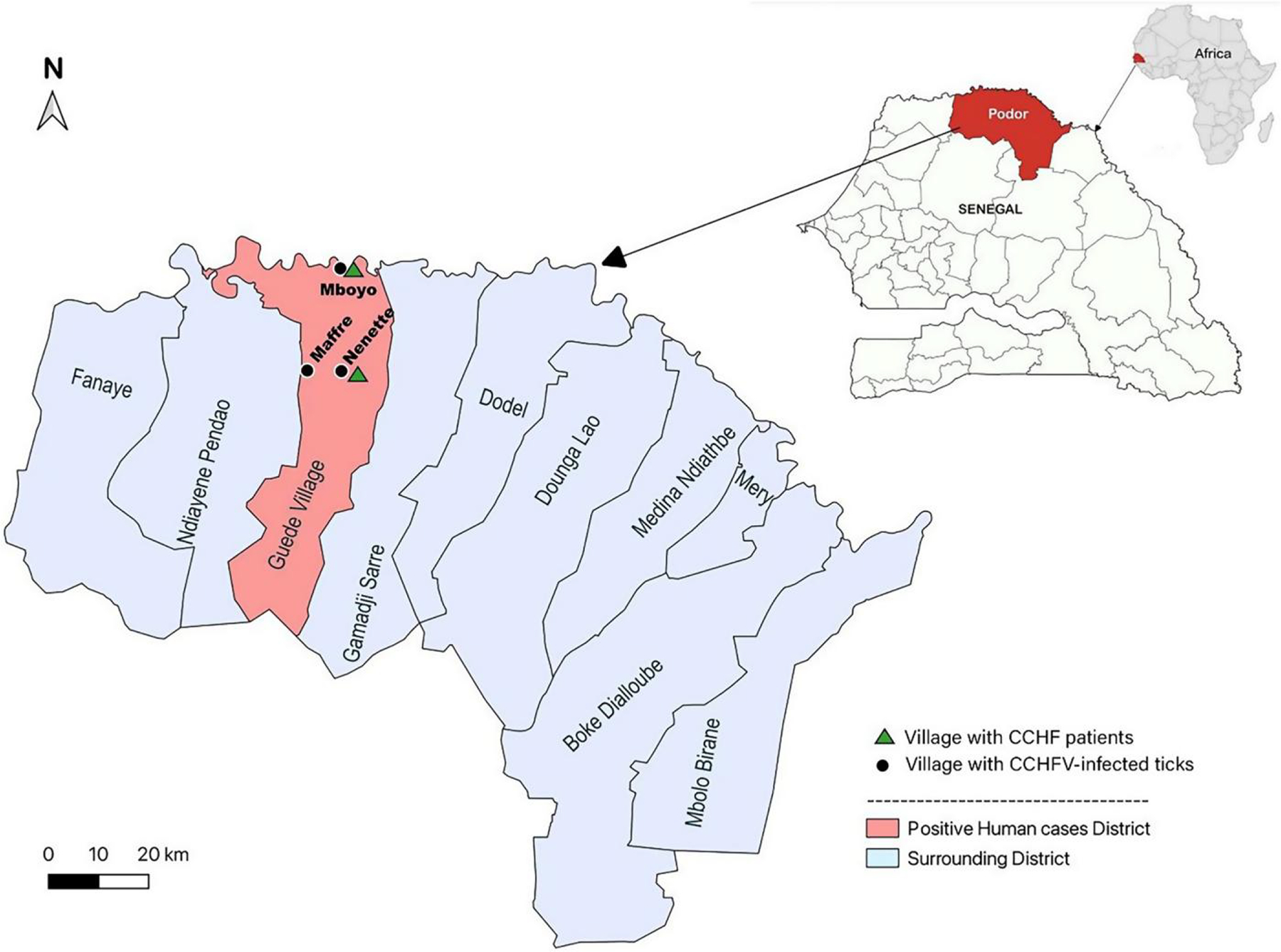
Map of Guede Village in Podor with positive human cases. CCHF = Crimean–Congo hemorrhagic fever; CCHFV = Crimean–Congo hemorrhagic fever virus.

The study was conducted in Podor, Saint-Louis region (16°30′00″N, 14°45′00″W) bordered by the Islamic Republic of Mauritania to the north, the district of Pété to the east, Linguere and Dahra to the south, and Dagana to the west (Figure 1). Podor is an important agrosylvopastoral and halieutic department. Podor has a Sahelian-type climate marked by the presence of the Harmattan (a hot, dry, dust-laden wind) during the long dry season and a short rainy season (July to October). Agriculture, livestock, small trade, crafts trade, and transport are the main socioprofessional activities of the population.

### Presentation of the viral hemorrhagic fever surveillance system in Senegal.

The Institut Pasteur de Dakar (IPD) hosts a WHO collaborating center for arboviruses and hemorrhagic fever viruses that in collaboration with the Senegalese Ministry of Health (MoH), provides viral hemorrhagic fever (VHF) case detection. This surveillance is usually more active in regional hospitals, where any patient with a corrected axillary temperature ≥38°C associated with hemorrhagic manifestations (epistaxis, gingivorrhagia, metrorrhagia, etc.) is considered a VHF suspected case. Blood samples are systematically collected in 5-mL ethylenediamine tetraacetic acid tubes from the suspected cases and sent to the IPD for laboratory test of different, viruses including CCHFV.

### Outbreak investigations.

During this outbreak, epidemiological and zoological investigations were performed by the MoH in collaboration with IPD and other partners (like Laboratoire National de l’Elevage et de Recherches Vétérinaires and Institut Sénégalais de Recherches Agricoles) to monitor the patient’s viral load and seroconversion and identify additional human cases around the confirmed case and in the surrounding villages as well as the animal and arthropod infection sources.

### Human sample collection.

During the investigations in the village where the confirmed case was detected, any VHF suspected case meeting the case definition described above was sampled on the same days as the consultation in the health care center and followed up every 3 days to minimize the risk of misdiagnosis. In addition, all contact cases, defined as anyone living in contact with the confirmed or suspected case, were listed, sampled on the same day, and then, monitored daily for 14 days. The investigation team maintained contact with them by mobile phone to monitor for any symptomatic signs of CCHF. During the follow-up, all contacts who develop signs of illness were sampled for laboratory testing. This protocol was also implemented in the communities neighboring the location of the confirmed cases and their respective villages to detect additional human cases. Blood samples from suspected and contact cases were collected in 5-mL ethylenediamine tetraacetic acid tubes and shipped to the IPD for laboratory testing.

### Domestic animal and tick samples collection.

Samples were collected only in areas with confirmed human cases. Animal sampling was performed by local veterinarians. During the outbreak investigations, blood samples and ticks were collected from 76 sheep, 90 goats, one equine, and 61 cattle found in villages of the confirmed cases and randomly selected surrounding villages.^23^ Animal blood samples and tick samples were collected in all areas as previously described.^23^

The ticks were then transported to the IPD laboratory in liquid nitrogen, speciated, and sorted in pools of 2–34 individuals composed of ticks of the same sex, stage, and species as well as collected from the same attachment sites, host, and location as previously described.^23–25^ These pools were stored at −80°C until further analyses. A total of 360 mono-specific pools were obtained with a predominance of *Hyalomma impeltatum* and *Rhipicephalus evertsi*, known vectors of and often found associated with the CCHFV in Senegal.^20,21,26^

### Sample processing.

#### Human sample testing.

Virological analyses (real-time reverse transcription polymerase chain reaction (RT-PCR) and IgM and IgG ELISA) for several viruses, including CCHF, Rift Valley fever, yellow fever, and dengue viruses, were performed for the samples of the VHF suspected cases and of the contact cases who developed symptoms. In addition, these suspected VHF samples were also tested for Ebola, Marburg, and Lassa viruses by RT-PCR. One hundred and forty microliters of clinical sample was used for RNA extraction using the QiaAmp Viral RNA Mini Kit according to the manufacturer’s instructions (Qiagen, Hilden, Germany). The lightMix kit (TIB Molbiol, Berlin, Germany), primers, and probe previously described were used for RT-PCR.^27^ Serological testing for detection of CCHFV IgM and IgG was also performed on human samples by using IPD in-house ELISAs. Indeed, in-house ELISAs have been developed at the IPD by using mouse-produced antigens and immune ascites for different arboviruses, including CCHFV.^28^

#### Animal and tick samples testing.

Each of the tick pools was placed in a 2-mL tube containing three metal beads and 1 mL of cell culture medium consisting of L-15 (Gibco BRL, Grand Island, NY, USA) and 20% cold fetal bovine serum. This mixture was automatically ground using the Tissue Lyser (Qiagen) for 2 minutes at a frequency of 30 Hz/second and then, centrifuged at 10,000 × *g* at +4°C for 5 minutes. After centrifugation, the supernatant was filtered using a 0.20-*μ*m filter (Sartorius, Göttingen, Germany), collected in a CryoTube (Thermo Fisher Scientific, Waltham, MA) using a 1-mL syringe (Artsana, Como, Italy) and stored at −80°C.

Subsequently, 140 *μ*L were used for each blood and tick sample for RNA extraction using the QiaAmp Viral RNA mini kit, and CCHF RT-PCR was conducted using the method previously described. Antibody (IgM/IgG) against CCHFV detection in animal sera was performed using ID Screen CCHF Double Antigen Multispecies (IDvet, Grabels, France) following the manufacturer’s instruction.^29^

### Viral genome sequencing and phylogenetic analyses.

Sequencing was performed using the RNA extracted from the CCHF RT-PCR-positive samples as previously described.^30^ Briefly, host ribosomal RNA was depleted using specific probes and Oligo-dT. The depletion mixture underwent heat denaturation at 95°C for 2 minutes followed by slow cooling to 45°C at a rate of 0.1°C/second. Subsequently, RNA hybrids were degraded using RNase H (NEB, Hitchin, United Kingdom) followed by treatment with TURBO DNase (Thermo Fisher Scientific) and purification using RNAClean XP beads (Beckman Coulter, Nyon, Switzerland). The depleted RNA served as a template for first-stranded complementary DNA (cDNA) synthesis using the SuperScript IV Reverse Transcriptase kit (Invitrogen, Thermo Fisher Scientific). The double-stranded cDNA was generated using the Klenow exo-DNA polymerase (NEB). Whole-genome sequencing was performed using a hybrid capture approach with the Twist Biosciences Comprehensive Viral Research Panel. Briefly, cDNA was fragmented before a telomere repair followed by dA-Tailing and a ligation step with Universal Twist adapters before final library amplification. A single library pool was finally prepared from the indexed library-prepared samples and then, subjected to target hybridization in solution followed by binding of the hybridized targets to the desired streptavidin beads. Enriched sample libraries obtained were loaded onto an Illumina MiSeq v. 3 (150 cycles) instrument (San Diego, CA).

The sequencing reads were analyzed using the EDGE bioinformatics pipeline (https://www.edgebioinformatics.org/; accessed January 5, 2022), which generated a single Fasta file for each sample. The assembled sequences of the S, M, and L segments were submitted to Genbank. Nearly complete genomes obtained were submitted to the public database basic local alignment search tool to identify their homologous sequences and then, combined with a representative subset of CCHFV sequences available in Genbank. Multiple sequence alignment was performed using the MAFFT program.^31^ The maximum likelihood (ML) phylogenetic trees for the three genome segments were inferred with the IQ-TREE software. An automatic model selection argument was applied using a model finder implemented in the software based on the Bayesian Information Criterion with 1,000 bootstrap replicates.^32^

## RESULTS

### Human CCHF cases.

#### Confirmed case 1: First index case.

On August 2, 2022, a 43-year-old female patient residing in the village of Mboyo presented to the Mboyo health post (Podor department, Saint-Louis region; 16°38′56″N, 14°57′38″W) with a febrile syndrome, headache, vertigo, arthralgia, myalgia, gingivorrhagia, and a negative malaria rapid diagnostic test (RDT). The symptom onset dated back to 14 days before her admission. The patient reported having direct contact with livestock (cows, sheep, goats, donkeys, horses) living in her household. She also reported a history of travel to Mauritania on July 12. She was treated with a single dose of injectable novalgin 1 g and ampicillin 500 mg every 6 hours, and then, she was placed under observation for 12 hours. After that, she was discharged with a prescription for ciprofloxacin 500 mg every 12 hours and paracetamol 1,000 mg every 6 hours. On August 5, 2022, she came back to the Mboyo health post for persistent gingival bleeding and was transferred to the Podor Health Center, where she developed pain syndrome, anemic syndrome, gingivorrhagia, subicterus, allegation fever, and cardiovascular collapse. On day 3 of hospitalization (August 7), the patient died of hemorrhagic shock (gingivorrhagia, metrorrhagia). Treated as a case of suspected VHF, a post-mortem blood sample was taken and sent to the IPD as part of the VHF surveillance on August 8. The sample was positive for CCHFV by RT-PCR and by IgM ELISA. Subsequently, an outbreak investigation team was deployed to the area by the MoH on August 12.

#### Confirmed case 2: Contact case of the first suspected case.

On August 10, 2022, a 79-year-old woman who had direct contact with the index case and was residing in the same household presented to the Podor Health Center for febrile syndrome, cough, physical asthenia, arthralgia, and myalgia. The date of the disease onset was August 9. On examination, she presented with fever (*T* = 40°C), rhonchi noted in both lung fields, infectious syndrome, pain syndrome, and a negative malaria RDT. She was hospitalized and treated with injectable ceftriaxone 2 g every 12 hours, injectable vitagen 2 mL every 12 hours, injectable tramadol 1,000 mg, vitamin C 1,000 mg once daily (morning), paracetamol 1,000 mg every 12 hours, and iron 1 mg every 24 hours. Because of the persistence of the symptoms, the appearance of other suspicious signs (abdominal pain, dizziness, apyrexia), and confirmation of the CCHF index case, a blood sample was taken on day 5 of the hospitalization (August 13) and sent to the IPD by the investigation team. The sample was positive by RT-PCR for CCHFV. A second blood sample was taken on day 19 of hospitalization and was negative by RT-PCR and positive by IgM ELISA for CCHFV. The patient recovered without complications and was discharged on August 9.

#### Confirmed case 3.

Case 3 was a 62-year-old resident of Nenette (Béléle Kélé) who had a history of contact with livestock on her property and had recently returned from a trip to Mboyo. She presented on admission to the Podor Health Center on August 27 with a febrile syndrome (*T* = 38°C), negative malaria RDT, abdominal pain, headaches, arthralgia, myalgia, infectious syndrome, conjunctival jaundice, and a hemorrhagic syndrome (gingivorrhagia, hematuria). The onset of the symptoms dated back to August 23. She was hospitalized and initially treated with gelofusine, injectable ceftriaxone 2 g every 12 hours, and injectable azantac 50 mg every 8 hours, and she was then placed under observation. Because of the ongoing investigation in Mboyo, a blood sample was taken and sent to the IPD the same day. The sample was found to be positive by RT-PCR for CCHF. The patient died on day 4 of hospitalization (August 30) after hemorrhagic shock (gingivorrhagia, hematemesis, metrorrhagia, rectorrhagia).

### Detection of CCHFV-specific antibodies in human samples collected during the outbreak investigations.

During the investigations of the three confirmed cases, a total of 225 human samples were analyzed, but no CCHFV RT-PCR- or IgM-positive samples were detected. Among the 225 samples, 10 (4.4%) had detectable IgG antibodies against CCHFV. Notably, we observed varying levels of CCHFV IgG seropositivity, with the highest seroprevalence found in the Alana, Kadione, Biddi, Gaode Boffe, and Nenette villages. In Mboyo itself, no IgG-positive samples were detected. Detailed results are shown in Table 1.

### Detection of CCHFV antibodies in livestock.

All animal samples were negative for CCHFV RT-PCR. However, an overall seroprevalence (IgM/IgG) for CCHFV of 42.5% was observed among the tested animal sera. Differences in seroprevalence between animal species were observed (Table 2). Indeed, cattle showed the highest seroprevalence (83.3%) followed by goats (32.2%) and sheep (22.36%). The only equine sample tested was positive for CCHFV IgG.

### Detection of CCHFV in ticks.

Of 360 tick mono-specific pools tested, 11 were positive by RT-PCR for CCHFV. Seven were from the Mboyo village, three from the Nenette village, and one was from neighboring Maffre village. The highest positive rate for CCHFV was found in *H. impeltatum* ticks. Detailed results are shown in Table 3.

### Viral genome analysis and phylogenetic studies.

In this study, 12 CCHFV genome sequences were determined from human samples (3) and from ticks (9). The nucleotide sequences of all of the segments were deposited in GenBank (Table 4). Basic local alignment search tool for nucleotides analyses revealed that most of our isolates shared a very high nucleotide sequence similarity (over 98%) with strains isolated from Sudan in 2009 (AEI70585_SDN_2009), Spain in 2016 (ATG31912_SPN_2016), Nigeria in 2012 (APB09909_NIG_2012), and Mauritania in 1984 (ABB30015_MRT_1984). The ARD407242_SEN_2022 strain sequence, however, shared a higher identity (more than 97%) with the sequences of isolates from Senegal in 1969 (ABB30013_SEN_1969), 1972 (ABB30014_SEN_1972), and 1975 (WAD86873_SEN_1975) and from Mauritania in 1984 (ABB30015_MRT_1984).

**Figure 2.**
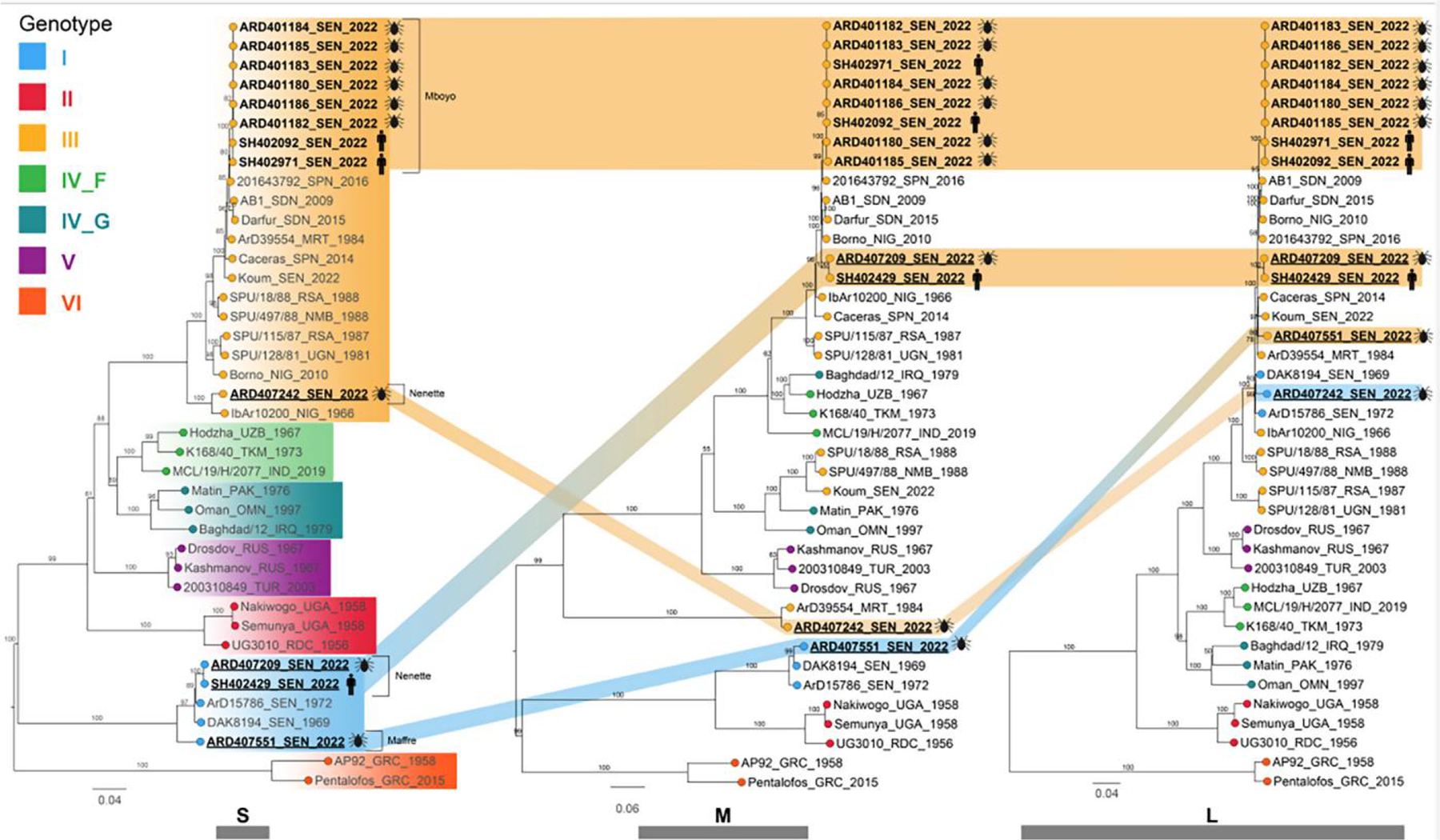
Phylogenetic analyses for the short (S), medium (M), and long (L) segments using recent Crimean–Congo hemorrhagic fever virus classification.

Phylogenetic analyses were performed for the S, M, and L segments using recent CCHFV classification^3^ (Figure 2). The analyses revealed the cocirculation of two distinct strains responsible for the human CCHF cases and extensive genetic diversity with evidence of virus reassortment. The first strain encompasses all genomes sampled in Mboyo village (two from humans and six from ticks), which consistently clustered within genotype III in all three segments, forming a monophyletic subclade along with strains isolated in Spain (2016) and Sudan (2009). In contrast, the second strain comprised genomes sampled in Nenette village (one from a human and one from ticks), with genotype I in the S segment clustering with strains isolated in Senegal in 1969 and 1972 (ABB30014_SEN_1972) and genotype III in the L and M segments, forming a distinct subclade separate from the Mboyo strains. Two additional strains with distinct genetic patterns sampled from ticks in the neighboring villages of Nenette and Maffre were determined (Figures 1 and 2). One strain sampled in Nenette shared the same genotype III as the Mboyo strains in the S and M segments but clustered within genotype I in the L segment. Another strain, identified in Maffre village, consisted of genotype I in the S and M segments and genotype III in the L segment. The ML tree incongruencies for the CCHFV strains from Nenette and Maffre suggest that these strains are reassortants of genotype I and III strains.

## DISCUSSION

Our study reported three human CCHF cases with two deaths in Podor, Saint-Louis region (northern Senegal) in 2022 and evidence of CCHFV infection in livestock and ticks. To our knowledge, this is the highest number of confirmed cases as well as deaths in a single outbreak in Senegal. Northern Senegal, a sylvopastoral area at the border with Mauritania with a record of CCHF outbreaks, has always been designated as a high-risk area of CCHF emergence.^22,33^ Indeed, most of the CCHFV strains from Senegal have been identified and isolated from this part of the country.^16,17,22^ In addition, different tick species and a high CCHFV seroprevalence in domestic animals have been reported from this area.^22,34^ So far, only very few human nonsevere and sporadic CCHF cases had been reported in northern Senegal before 2022.^16,17^ It is quite possible that CCHF asymptomatic or nonsevere CCHF cases were underreported because of inappropriate surveillance. The CCHFV IgG antibodies detected in humans in previous studies as well as in our study indicated that the disease might have been prevalent for longer in the area.^34–36^ However, this first severe appearance of CCHF human cases including mortalities is probably the result of the increasing prevalence of the virus in the area, the high abundance of animal hosts in this sylvopastoral zone, and the diversity of tick populations including the main CCHF vectors. Variations of these parameters could explain the different CCHF epidemic profiles in endemic countries.^37^ Thus, these data suggest that the conditions in northern Senegal have become favorable for the emergence and spread of CCHF and urgently require more studies to better understand the transmission dynamics of the virus and to strengthen surveillance for rapid detection and implementation of control strategies.

Since 2015, a sentinel syndromic surveillance (4S network) of arboviruses and VHFs in humans has been implemented by the MoH in many health districts of Senegal. The health care workers in the sentinel sites are well trained on arbovirus and VHF disease transmission, risk factors, and symptoms, which allowed early diagnosis of human cases and rapid responses.^38,39^ The 4S network detected two human CCHF cases in northern Senegal (Matam region); fortunately, both recovered from the infection in 2019 and 2022.^16,17^ However, there was no 4S sentinel site in Podor before 2022. During the outbreak, the two deceased patients had the particularity of presenting a severe disease with hemorrhagic syndrome (gingivorrhagia and metrorrhagia) in addition to the common disease signs (fever and myalgia), suggesting their late confirmation in the health facilities in Podor. During the assessment of the index case at the health post in Mboyo village, the suspicion for VHF was not raised despite the presence of bleeding, regular contact with livestock, and travel history to CCHF-endemic Mauritania. This situation in Podor confirmed the lack of training on VHFs of the health care workers in some areas in Senegal and after the outbreak, led to the extension of the 4S network by the MoH with inclusion of more sentinel sites, including the Podor district. Senegal does not have a national treatment guideline for CCHF infection. The treatment of CCHF is mainly symptomatic, which makes early detection of cases, as done in the 4S network, crucial for patient outcomes.

The outbreak investigations showed an overall CCHFV IgG seroprevalence of 4.4% in humans around the confirmed cases, varying between 0% and 100% across the villages, similar to seroprevalence data recently described in Matam, another region of northern Senegal.^34^ High seroprevalence in many villages including Nenette, where a CCHF case was detected, confirm that the virus is endemic in this area. No seroprevalence was detected in Mboyo village where two CCHF cases occurred, suggesting recent emergence of the virus in the area. Further studies of a larger representative population would be necessary to confirm this observation.

The high seroprevalence observed with cattle (83.3%) is probably because of their longevity and the long persistence of IgG antibodies. Seroprevalence data in cattle and goats from our study are higher than those recently reported in the north of the country by Mangombi et al.^22^ and similar to those reported in Mauritania.^40^ Our data indicate that livestock play a crucial role as reservoir hosts for CCHFV, contributing to the outbreak in the area. The only equine specimen tested in this study also showed IgG positivity for CCHFV. Further studies with bigger sample sizes are needed to fully understand the potential role of equines in CCHFV transmission. The MoH has now issued recommendations to avoid any direct contact with animal fluids and tissues.

Eleven CCHFV strains were isolated from in Mboyo, Nenette, and Maffre during the outbreak, mainly from *R. evertsi*, *H. impeltatum*, and *Hyalomma marginatum rifupes*. *R. evertsi* and *H. marginatum rifupes* are two species known to be maintenance vectors of CCHFV in the Sahelian zone.^26^ In addition, *H. marginatum rifupes*, *H. impeltatum*, and *R. evertsi* are competent vectors and were already associated with CCHFV in Senegal.^20^ In response to this outbreak, the National Hygiene Service organized a tick-spraying campaign to limit the risk of CCHFV infection through tick bites. The MoH also issued recommendations to the population, including regularly cleaning animals, using pliers to remove ticks attached to animals, and inspecting yourself after contact with animals.

In this outbreak, two of the three confirmed cases had regular exposure to CCHF IgG-positive livestock that were also infested with ticks containing CCHFV, confirming regular contact with livestock as a main transmission mode in the area as already described.^6^ The second CCHF case detected in the Mboyo village declared having assisted the index case during her illness as they lived in the same house. This secondary infection case is very likely because of the well-known human to human transmission mode of CCHF,^6^ and to our knowledge, this is the first CCHF secondary case detected in Senegal. However, vector-borne transmission for the second case remains plausible, considering the possibility that the same tick may have taken multiple successive blood meals first from the index case and then, from the second case. This could involve either an initially infected tick or another tick that became infected after feeding on the index case. Taken together, it is necessary to raise awareness among the population to limit the emergence and propagation of CCHFV in high-risk areas in Senegal.

The phylogenetic analysis of the 12 CCHFV genomes established an epidemiological link between the human and tick strains detected in Mboyo and Nenette villages, and it revealed that the Podor epidemic resulted from the independent emergence of two distinct CCHFV strains. The isolates from Mboyo belong to genotype III and are most closely related to isolates from Spain and Sudan, whereas the CCHFV genomes from Nenette belong to genotypes I and III, consistent with at least two independent introductions. The detection of distinct viral genotypes in both human and tick populations confirmed the crucial role of ticks in maintaining and transmitting different CCHFV strains in northern Senegal. Two more strains were also identified from ticks sampled in Nenette and Maffre, neighboring villages of Mboyo. The CCHFV genome from Maffre is related to viruses identified in Senegal in 1969 and 1972 and all across its entire tripartite genome, suggesting the long-term persistence of this genotype in northern Senegal. The genetic patterns of these two new CCHFV included inversely reassorted group (I and III) segments, and they appear to be reassortants between parent CCHFV strains belonging to genotypes I and III. The extraordinary diversity of CCHFV with four cocirculation distinct genome patterns, including multiple reassortments, indicates dynamic circulation and evolution of CCHFV in the respective reservoir host of this sylvopastoral region. Sylvopastoral systems have been introduced to Senegal to reverse deforestation.^41^ They are known to increase overall species diversity and have been described to increase invertebrate species diversity.^42^ It remains to be studied if the drive for sylvopastoral systems could be connected to the emergence of CCHFV. Changes of agricultural techniques are suspected to have contributed to the massive CCHFV emergence in the Tokat valley of Turkey.^43^ The reassortant genomes reported here indicated exchanges of genomic segments between viruses reported from various countries of West Africa, with the closest relatives often sampled more than 40 years ago. Reassortant CCHFV genomes were previously described in eastern Senegal in 2022.^17,44^ Reassortment in Bunyaviruses can increase their virulence as demonstrated by Ngari virus, a reassortant Orthobunyavirus with L and S segments from Bunyamwera virus and the M segment from Batai virus. Although Bunyamwera and Batai viruses typically cause moderate disease, Ngari has been linked to hemorrhagic fever cases in East Africa.^45,46^ Therefore, it would be essential to conduct phenotypic/clinical characterizations of these reassortants to assess if they are associated with particular pathogenicity. Despite the high CCHFV genetic variability in northern Senegal, the study also yielded evidence for the long persistence of older Senegalese CCHFV strains carried in *H. impeltatum* ticks in this area. The study also showed that the CCHFV strains detected in northern Senegal shared closed phylogenetic relationships with strains from other countries in Africa, such as Mauritania, Sudan, and Nigeria, as well as countries in Europe, such as Spain. Introduction of CCHFV strains through livestock and the migration of tick-infested birds from these countries may play a role here. Particularly, the border between Senegal and Mauritanian is porous for the entry of livestock potentially carrying infested ticks into northern Senegal.

Taken together, our data showed that the lack of an appropriate surveillance system prevented the timely detection of CCHF confirmed cases in Podor. Our data also suggest that the main mode of transmission of the virus is through contact with infected livestock and/or ticks. We also showed a high genetic diversity for CCHFV strains detected in Podor and suggest that the epidemic is owing to two different strains with different origins while identifying an unexpected diversity of reassortant strains in one human case in reservoir ticks. Finally, our study showed that carrying out targeted investigations around confirmed cases yields data on CCHFV circulation in reservoir hosts and vectors and will be crucial for better estimation of the disease burden and for more efficient responses.

## CONCLUSION

Northern Senegal is an area at high risk for CCHF emergence, and the recent implementation of the new 4S sentinel site will contribute to strengthening of the surveillance for CCHF as well as for other arboviral disease in humans. Sentinel veterinary care should also be implemented in the area for animals and ticks. Seroprevalence studies in livestock and high- and low-risk populations should also be conducted to better understand the transmission dynamics of CCHFV in the area and identify the risk factors to implement efficient control strategies.

## Figures and Tables

**Table 1:** Seroprevalence (IgG antibodies) of CCHFV in humans from the different villages in Podor from September to October 2022

Village	No. of Participants	CCHFV IgG Positive	Seroprevalence (%)
Nenette*	40	4	10.0
Mboyo*	17	0	0.0
Belel Kelle	9	2	22.2
Alana	1	1	100
Gaode Boffe	8	1	12.5
Biddi	6	1	16.7
Kadione	2	1	50.0
Maffre	7	0	0.0
Others	135	0	0.0
Total	225	10	4.4

CCHFV = Crimean–Congo hemorrhagic fever virus.

* Human cases were detected in these villages.

**Table 2:** Seroprevalence (IgM/IgG antibodies) of CCHFV in animals collected in the neighborhood of the human confirmed cases

Species	No. of Samples	CCHFV Positive (%)
Cattle	61	50 (83.3)
Goats	90	29 (32.2)
Sheep	76	17 (22.4)
Equine	1	1 (100)
Total	228	97 (42.5)

CCHFV = Crimean–Congo hemorrhagic fever virus.

**Table 3:** Detection of CCHFV in ticks collected in the neighborhood of the human confirmed cases

Genus	Species	No. of Ticks	No. of Pools	CCHFV-Positive Pools
*Hyalomma*	*impeltatum*	464	109	4
	*marginatum rifupes*	104	50	1
*Rhipicephalus*	*evertsi*	379	143	2
	*senegalensis*	10	3	2
	*sulcatus*	5	2	2
Others*	–	86	53	0
Total	–	1,048	360	11

CCHFV = Crimean–Congo hemorrhagic fever virus.

* Others include *Hyalomma truncatum*, *Rhipicephalis guilhoni*, *Rhipicephalis muhsamae*, and *Boophilus decolaratus*.

**Table 4:** Strains of CCHFV isolated in this study, including GenBank accessions numbers for each segment, their respective hosts species, and areas of origin

			Segment Accession Nos.		
CCHFV Strains	Host	Village	L	M	S
SH401971_SEN_2022	Patient 1	Mboyo	PP835511	PP894725	PP894733
SH402092_SEN_2022	Patient 2	Mboyo	PP845328	PP894728	PP894735
SH402429_SEN_2022	Patient 3	Nenette	PP835508	PP894722	PP894730
ARD401180_SEN_2022	*Rhipicephalus senegalensis*	Mboyo	PP894721	PP894745	PP894740
ARD401182_SEN_2022	*Rhipicephalus sulcatus*	Mboyo	PP894720	PP894744	PP894736
ARD401183_SEN_2022	*Rhipicephalus evertsi*	Mboyo	PP894719	PP894743	PP894737
ARD401184_SEN_2022	*Rhipicephalus evertsi*	Mboyo	PP835510	PP894726	PP894732
ARD401185_SEN_2022	*Hyalomma marginatum rifupes*	Mboyo	PP916049	PP894742	PP894739
ARD401186_SEN_2022	*Rhipicephalus sulcatus*	Mboyo	PP894718	PP894741	PP894738
ARD407209_SEN_2022	*Hyalomma impeltatum*	Nenette	PP835507	PP894724	PP894729
ARD407242_SEN_2022	*Hyalomma impeltatum*	Nenette	PP835512	PP894727	PP894734
ARD407551_SEN_2022	*Hyalomma impeltatum*	Maffre	PP835509	PP894723	PP894731

CCHFV = Crimean–Congo hemorrhagic fever virus; L = long; M = medium; S = short.
